# Build-To-Specification Vanillin and Phloroglucinol Derived Biobased Epoxy-Amine Vitrimers

**DOI:** 10.3390/polym12112645

**Published:** 2020-11-10

**Authors:** Aratz Genua, Sarah Montes, Itxaso Azcune, Alaitz Rekondo, Samuel Malburet, Bénédicte Daydé-Cazals, Alain Graillot

**Affiliations:** 1 CIDETEC, Basque Research and Technology Alliance (BRTA), Paseo Miramón, 196, 20014 Donostia-San Sebastián, Spain; smontes@cidetec.es (S.M.); iazcune@cidetec.es (I.A.); arekondo@cidetec.es (A.R.); 2 SPECIFIC POLYMERS, Zac Via Domita, 150 Avenue des Cocardières, 34160 Castries, France; samuel.malburet@specificpolymers.fr (S.M.); benedicte.dayde-cazals@specificpolymers.fr (B.D.-C.); alain.graillot@specificpolymers.fr (A.G.)

**Keywords:** biobased epoxy, vitrimers, recyclable thermosets

## Abstract

Epoxy resins are widely used in the composite industry due to their dimensional stability, chemical resistance, and thermo-mechanical properties. However, these thermoset resins have important drawbacks. (i) The vast majority of epoxy matrices are based on non-renewable fossil-derived materials, and (ii) the highly cross-linked molecular architecture hinders their reprocessing, repairing, and recycling. In this paper, those two aspects are addressed by combining novel biobased epoxy monomers derived from renewable resources and dynamic crosslinks. Vanillin (lignin) and phloroglucinol (sugar bioconversion) precursors have been used to develop bi- and tri-functional epoxy monomers, diglycidyl ether of vanillyl alcohol (DGEVA) and phloroglucinol triepoxy (PHTE) respectively. Additionally, reversible covalent bonds have been incorporated in the network by using an aromatic disulfide-based diamine hardener. Four epoxy matrices with different ratios of epoxy monomers (DGEVA/PHTE wt%: 100/0, 60/40, 40/60, and 0/100) were developed and fully characterized in terms of thermal and mechanical properties. We demonstrate that their performances are comparable to those of commonly used fossil fuel-based epoxy thermosets with additional advanced reprocessing functionalities.

## 1. Introduction

Epoxy thermosets are widely used as matrices in lightweight fibre-reinforced composites for highly demanding sectors, such as, construction and transport. Epoxy-based composites are appreciated due to their dimensional stability, chemical resistance, and favorable thermo-mechanical properties arising from their crosslinked molecular structure. Most popular epoxy networks are based on bisphenol A (BPA) since the aromatic ring confers good thermal and mechanical properties. However, there are several social and environmental drawbacks associated with them: (i) traditional epoxy monomers, such as diglycidyl ether of bisphenol A (DGEBA), are manufactured from non-renewable fossil-derived precursors contributing to global CO_2_ emissions, exhaustion of natural resources and oil dependency, (ii) some base chemicals, such as BPA, are toxic substances, and (iii) the crosslinked thermosets cannot be efficiently repaired or reprocessed into new shapes which limits their end-of-life treatment and recycling possibilities [1]. Therefore, new strategies to move towards a more sustainable and competitive composite industry, also from the circular economy perspective, are highly demanded. In this paper, we focus on two strategies to face these limitations which consist of (i) using new monomers derived from renewable feedstock as replacement of traditional monomers and (ii) incorporating reversible crosslinks to turn the polymer matrices into covalent adaptable networks (CANs) [2,3,4,5,6].

The chemical industry is looking for aromatic alternatives to replace BPA and develop fully or partially biobased materials [7,8]. In this context, aromatic derivatives of biomass have been explored such as vanillin and phloroglucynol [9]. Vanillin is one of the few aromatic building-block that is industrially available from renewable resources, such as lignin [10]. Lignin is a highly cross-linked complex polymer built of substituted phenols and, along with cellulose and hemicellulose, the main component of the lignocellulosic biomass. Importantly, lignin is currently a by-product of the paper industry with high potential for valorisation. Vanillin can be turned into bi-functional epoxy monomer such as diglycidyl ether of vanillyl alcohol (DGEVA). This monomer has been already used for the preparation of cured epoxy resins [10,11,12,13,14]. Phloroglucinol is an aromatic product that occurs naturally in certain plants and marine species, notably in brown algae. Phloroglucinol was originally prepared from phloretin, a compound isolated from fruit trees. Moreover, phloroglucinol can be obtained by the bioconversion of sugar derivatives [15]. This compound can be functionalized with epoxy groups to turn it into a trifunctional epoxy monomer, phloroglucinol triepoxy (PHTE) [16].

Due to their bifunctional and trifunctional epoxy functionalities, DGEVA in combination with PHTE can be a very interesting resin mixture for the preparation of high-performance thermoset epoxy materials with tailored thermomechanical properties.

CANs can be considered to be on the border between thermosets and thermoplastics since they can change topology by thermally activated bond-exchange processes, and turn into dynamic networks under certain processing conditions. Depending on the exchange mechanism of the dynamic bonds, CANs are classified into associative and dissociative types. Associative CANs, also known as vitrimers, are based on chemical bonds that undergo exchange reaction while maintaining the crosslinking density throughout the process. On the contrary, dissociative CANs imply that the chemical bond is cleaved and reformed during the exchange process, leading to a viscosity drop during the reprocessing step. There are plenty of dynamic chemistries that lead to a plethora of materials [2,17,18]. In this regard, our group reported epoxy vitrimers based on the reversible aromatic disulfide linkages. The DGEBA epoxy matrix was used to manufacture carbon fibre-reinforced composites that are reprocessable, repairable and recyclable (3R) [19,20]. The aromatic disulfide crosslinks are easily incorporated in the epoxy network by readily available amine-terminated hardener, i.e., 4-aminophenyl disulfide (4-AFD).

Here, we present a second generation of aromatic disulfide-based epoxy vitrimers by starting from biobased vanillin and phloroglucinol precursors. The combination of those monomers and their compatibility makes a build-to-specification approach possible, since their thermal and mechanical properties can be tailored by changing the ratio of bifunctional DGEVA and trifunctional PHTE. Thus, four epoxy matrix formulations were developed combining different epoxy monomers’ ratio (DGEVA/PHTE wt%: 100/0, 60/40, 40/60, and 0/100) and 4-AFD. These matrices were fully characterized in terms of thermal, mechanical, and dynamic (reprocessability, repairability, and recyclability) properties.

## 2. Materials and Methods

### 2.1. Materials

**Figure 1:**
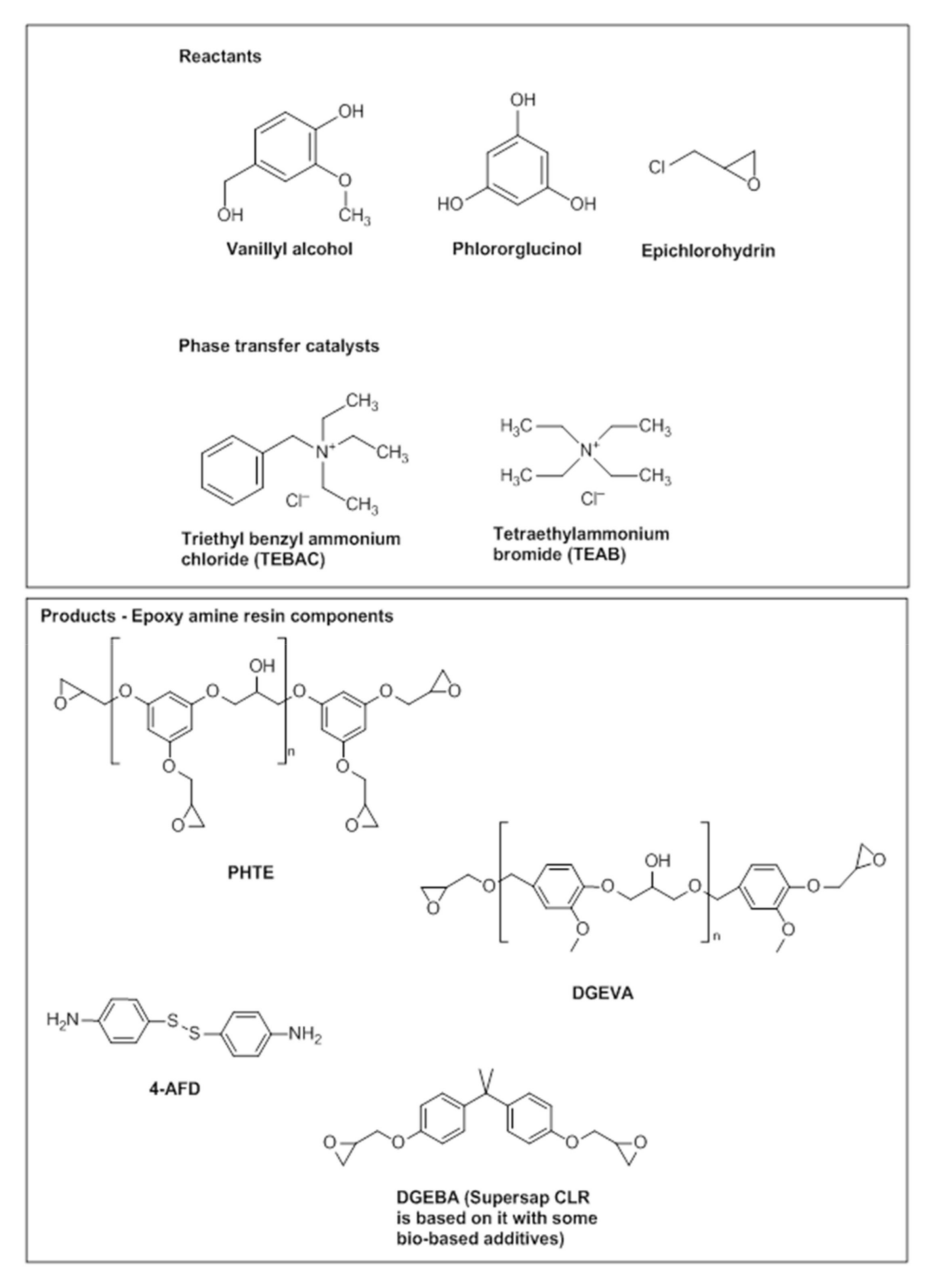
Structural formula of the used reactants and obtained products.

Briefly, 4-aminophenyl disulfide (4-AFD) was purchased from Molekula (Rimini, Italy). Vanillyl alcohol (98%), phloroglucinol (99%), epichlorohydrine (99%), benzyltriethylammonium chloride (TEBAC, 99%), anhydrous sodium sulfate (Na_2_SO_4_, 99%), sodium hydroxide pellets (NaOH), and all used solvents (ethyl acetate, dichloromethane) (>95%) were purchased from Sigma Aldrich (Saint-Quentin Fallavier, France). Tetraethylammonium bromide (TEAB, 98%) was purchased from Alfa Aesar (Kandel, Germany). Supersap CLR epoxy resin system was purchased from Entropy Resins (Cupertino, CA, USA). All reagents, reactants and solvents were used as received (Figure 1).

### 2.2. Methods

The ^1^H Nuclear magnetic resonance (NMR) spectra of the synthesized monomers were obtained using Bruker Advance 300 (300 MHz) spectrometer (Billerica, MA, USA( equipped with a QNP probe at room temperature. Deuterated solvents used are given for each molecule. The epoxide index (EI, number of moles of epoxide groups per gram, eq·g^−1^) was determined according to ^1^H NMR quantitative titration method (^1^H qNMR). The method consists in solubilizing a known mass of the product and of an internal standard (Benzoic acid (5 H equivalents) in acetone-d6 whose signal is dissociated from the others). The number of moles of epoxide functions per gram of product was measured by comparing the integration of the standard (5H) with the integration of a signal accounting for all the oxirane rings (for instance H4a, H4b, H8a and H8b for DGEVA, i.e., 4H).

The average number of repeating units (*n*) was also determined by ^1^H NMR, comparing the epoxide integrations (3H per phloroglucinol (H3) and 4H per DGEVA (H4a, H4b, H8a, H8b)) to aromatic integrations (3(2n + 1)H per molecule).

Fourier transform infrared (FTIR) spectra were recorded using a JASCO-4100 spectrometer from ThermoFisher scientific (Waltham, MA, USA) with a diamond ATR probe.

Thermal analysis was performed using a differential scanning calorimeter (DSC) instrument Discovery DSC25 Auto from TA Instruments (New Castle, DE, USA) over a temperature range from 25 to 220 °C under nitrogen. The glass transition temperature (T_g_) was obtained as the half-height of the heat flow step recorded at a scan rate of 20 °C/min.

Resins’ viscosities were measured with a Discovery Hybrid TA-Instrument (New Castle, DE, USA) rheometer (advanced Peltier plate—40 mm plate with a shear rate of 10 rad/s).

Thermal gravimetric analysis (TGA) measurements were performed in a TA Instruments Q500 equipment (New Castle, DE, USA) from 25 to 600 °C at a heating rate of 10 °C/min under nitrogen atmosphere.

Macroscopic scale reprocessing experiments were carried out using a VOGT hot LABO PRESS 200T (Lostorf, Switzerland). A film of the cured vitrimer was placed in a zig-zag shaped mould inside the press. The press was closed, and heat and pressure were applied onto the material for 10 min in order to reshape it.

Mechanical testing was performed using an INSTRON 3365 Long travel Elastomeric Extensometer (Norwood, MA, USA) controlled by Bluehill Lite software. Tensile strength measurements were carried out according to the ASTM D 638-08 standard, using type V test specimens and an elongation rate of 1 mm·min^−1^.

Dynamic mechanical analysis (DMA) was performed in a TA instruments Q800 (New Castle, DE, USA), using single cantilever (20 mm) clamps and at a heating rate of 3 °C/min in a temperature range from 25 to 220 °C. Stress-relaxation experiments were also carried out by DMA using 36 × 6 × 1.5 mm^3^. Samples were initially preloaded at a force of 1 × 10^−3^ N to maintain straightness. After reaching the testing temperature, samples were left for 5 min to reach the thermal equilibrium, then strained by 1%, and the deformation was maintained during the test.

A Pulverisette 19 equipment from Fritsch (Idar-Oberstein, Germany) (with a sieve size of 250 µm) was used for grinding the prepared thermosets for mechanical recycling.

### 2.3. Synthesis of Biobased Epoxy Resins and Vitrimers

#### 2.3.1. Synthesis of DGEVA

**Scheme 1:**
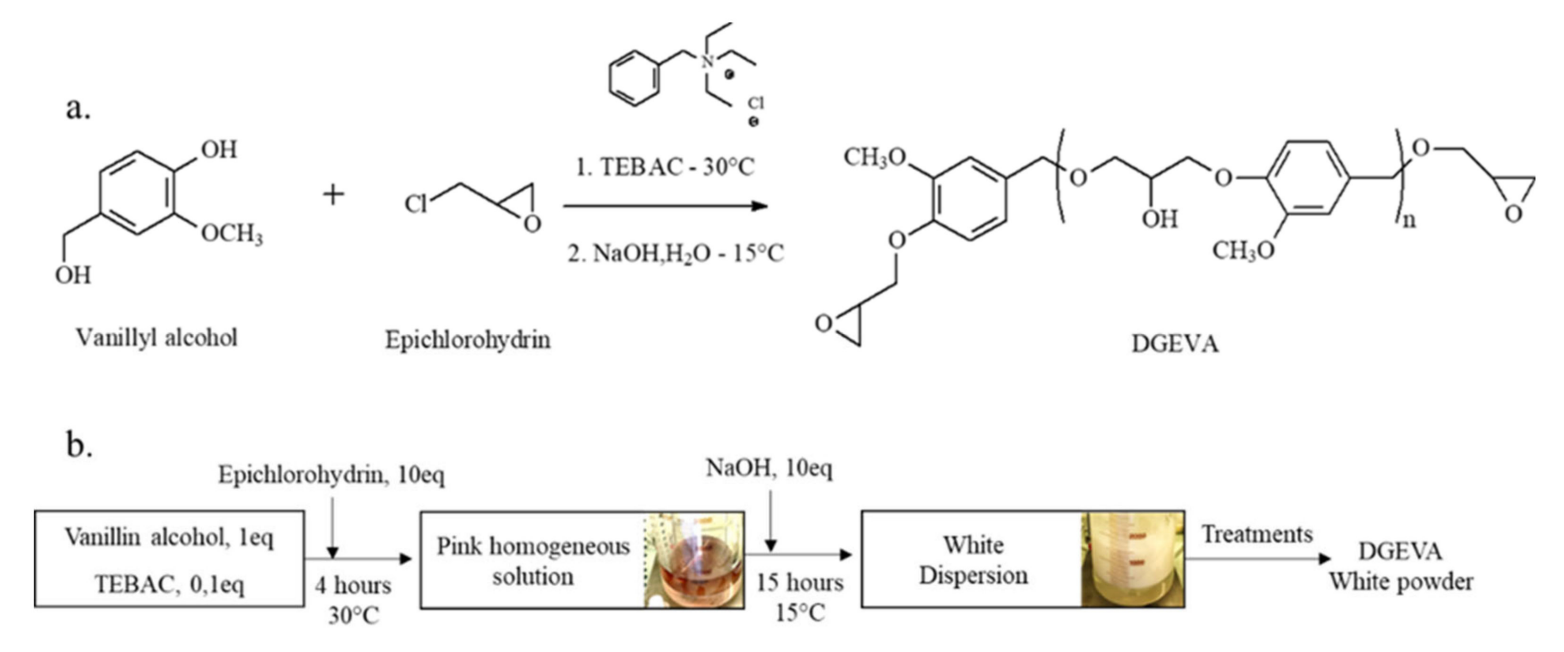
(**a**) Synthetic pathway; and (**b**) block diagram for the synthesis of DGEVA.

DGEVA was synthesized according to a two-step process as shown in Scheme 1 [16].

A three-necked reactor equipped with a thermometer and a mechanical stirrer was charged with vanillyl alcohol (100 g, 0.65 mol, 1.0 eq) and benzyltriethylammonium chloride (TEBAC, 15 g, 0.06 mol, 0.1 eq). Epichlorohydrin (600 g, 6.5 mol, 10.0 eq) was added and the mixture was stirred for 4 h at 30 °C until obtainment of a limpid pink solution. Then, this solution was cooled down to 15 °C. A NaOH solution (33 wt%, 15.0 eq.) was prepared and poured slowly into the cold mixture under vigorous stirring. The reaction was conducted overnight at 15 °C. Deionized water was added (600 mL) to the mixture as well as ethyl acetate (2000 mL). Then, the organic layer was washed two more times with deionized water and dried on anhydrous Na_2_SO_4_. Residual solvents were removed on a rotary evaporator at 60 °C. DGEVA (batch number SP-9S-5-005) was obtained as a white solid with a yield of 94% and the product was characterized by ^1^H NMR. Determination of the epoxy value was carried out by ^1^H qNMR. The epoxy index was evaluated at 7.28 meq·g^−1^ and the average-number of repeating units was estimated here at *n* = 0.03, which is in good agreement with the theoretical value of 7.51 meq·g^−1^. Corresponding molecular weight: 272.6 g·mol^−1^. Viscosity of DGEVA resin was measured at various temperatures (20 °C: 750 cP; 50 °C: 85 cP and 80 °C: 20 cP).

DGEVA: ^1^H NMR (300 MHz, CDCl_3_, ppm) δ: 2.64 (dd, 1H, H8b); 2.76 (m, 2H, H4b); 2.82 (m, 1H, H8a); 2.91 (m, 1H, H4a); 3.21 (m, 1H, H7); 3.43 (m, 2H, H6b, H3); 3.75 (dd, 1H, H6a); 3.90 (s, 3H, H9); 4.06 (dd, 1H, H2b); 4.24 (dd, 1H, H2a); 4.52 (d, 2H, H5); 6.90 (m, 3H, H1) (Supplementary Figure S1).

#### 2.3.2. Synthesis of PHTE

**Scheme 2:**
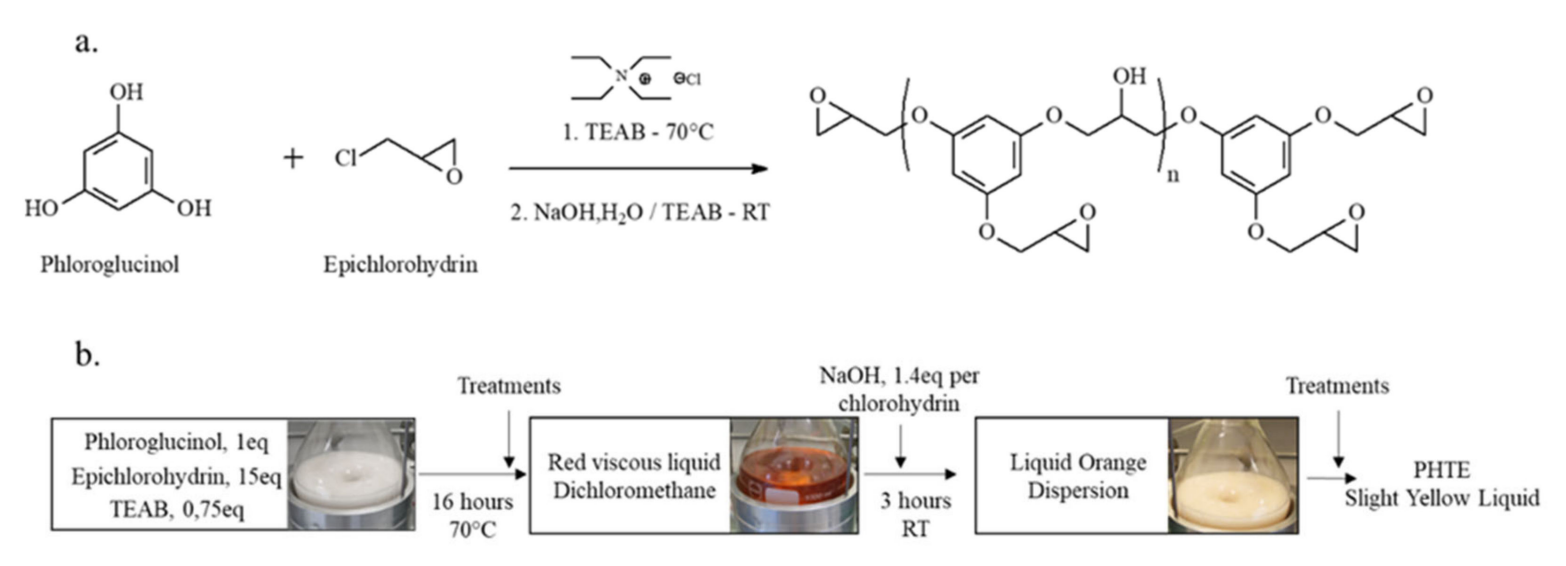
(**a**) Synthetic pathway; and (**b**) block diagram for the synthesis of PHTE.

PHTE was synthesized according to the synthesis pathway presented in Scheme 2 [16].

Phloroglucinol (50.0 g, 0.4 mol, 1 eq), epichlorohydrin (550.2 g, 5.95 mol, 15 eq), and tetraethylammonium bromide (TEAB, 62.5 g, 0.3 mol, 0.75 eq) were mixed in a 1 L three-necked reactor equipped with a thermometer, mechanical stirrer, and a condenser. The reaction mixture was stirred vigorously at 70 °C for 16 h. Then, the mixture was cooled down at room temperature and poured in dichloromethane (800 mL). The organic layer was washed three times with deionized water, dried on anhydrous Na_2_SO_4_, filtered, and concentrated on rotary evaporator. A red viscous liquid (116.6 g, 1.2 eq of chlorohydrin) was obtained and used in the second step without any further treatment. The red viscous liquid was solubilized in dichloromethane (1000 mL) and TEAB (4.17 g) was added to the mixture. Then, a NaOH solution (22 wt%, 1.4 eq. compared to residual chlorohydrin) was added dropwise for 30 min with continuous stirring. The reaction was carried out at room temperature for 3 h. Afterwards the mixture was washed three times with 200 mL of distilled water. The organic layer was dried on anhydrous Na_2_SO_4_ for 12 h and filtered. The excess solvents were removed under reduced pressure at 60 °C with a rotary evaporator. PHTE(batch number SP-9S-5-003) was obtained as a pale-yellow viscous liquid (111 g) with a yield of 95% and the product was characterized by ^1^H NMR. Determination of the epoxy value was carried out by ^1^H qNMR. The epoxy index was evaluated at 6.78 meq·g^−1^ and the average-number of repeating units was estimated here at *n* = 0.15. Theoretical value in this case was 10.2 meq·g^−1^. Deviations between theoretical and experimental epoxy values were explained in a previous paper [16]. Corresponding molecular weight: 330.0 g·mol^−1^. The viscosity of PHTE resin was measured at various temperatures (20 °C: 130 000 cP; 50 °C: 3 000 cP and 80 °C: 300 cP).

PHTE: ^1^H NMR (300 MHz, acetone-d6, ppm) δ: 2.70 (m, 3H, H4b); 2.84 (m, 3H, H4a); 3.30 (m, 3H, H3); 3.83 (dd, 3H, H2b); 4.28 (dd, 3H, H2a); 6.20 (s, 3H, H1) (Supplementary Figure S2).

#### 2.3.3. Synthesis of Epoxy-Vitrimers

Four different formulations were prepared varying the DGEVA/PHTE ratio (ECO-1 to ECO-4) with the aim of proving that the properties of the end material can be tailored as a function of this ratio. Additionally, a reference formulation (ECO-REF) was prepared using the biobased and commercially available Supersap CLR epoxy resin in combination with 4-AFD hardener (see Table 1). This resin consists of DGEBA (the most commonly used epoxy resin) with a biobased content that can go up to 25% and, thus, it was considered as a good reference for the developed formulations.

**Scheme 3:**
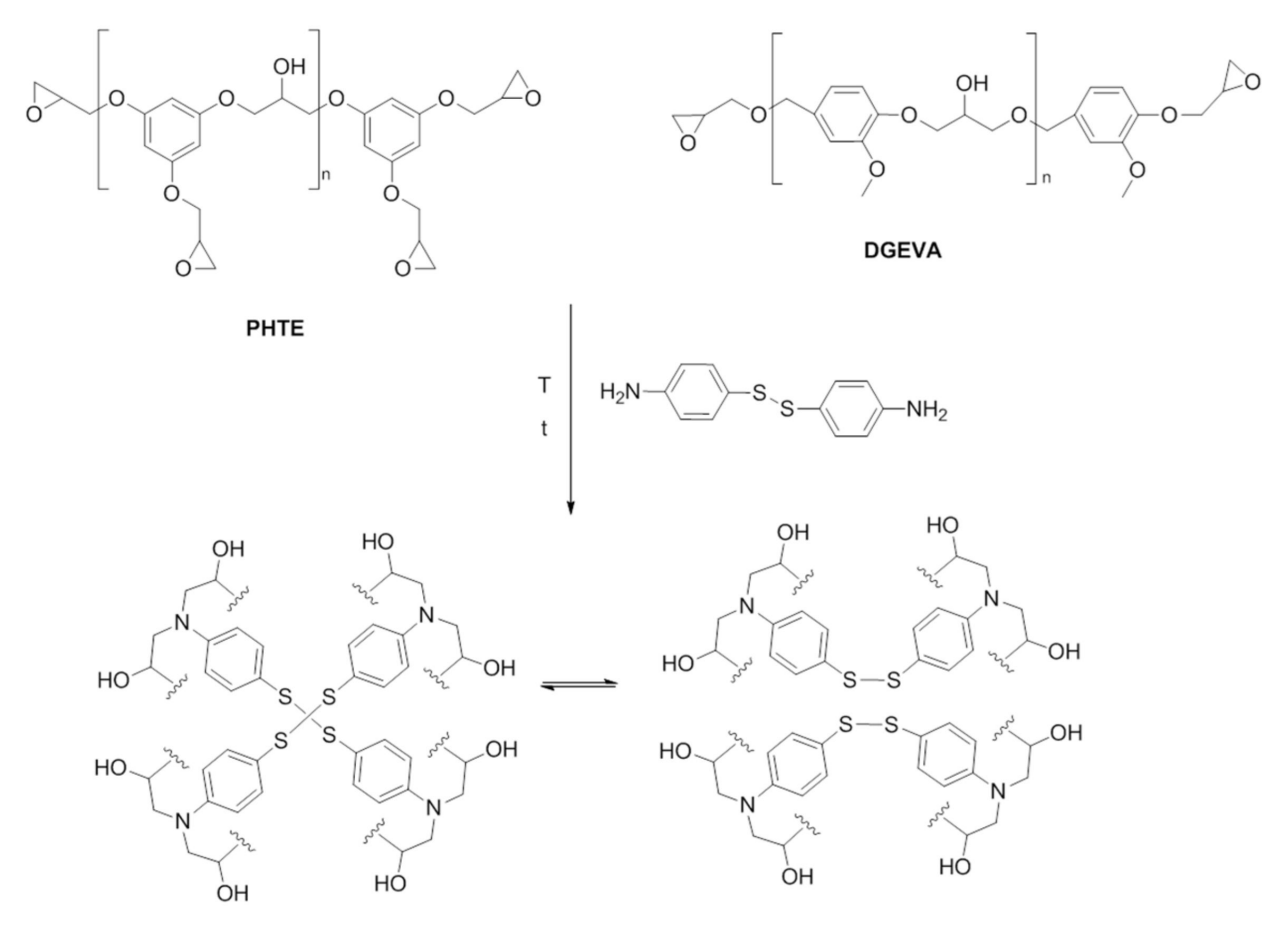
Synthetic route for epoxy-vitrimers. Required time and temperature are listed in Table 1.

All the formulations were prepared following the same procedure (Scheme 3). The corresponding resin mixture was first heated at 60 °C to reduce its viscosity and, afterwards, it was mixed with the hardener (previously melted at 80 °C). An excess of hardener was employed (1.2 amino eq. per 1 epoxy eq.) which allowed having extra aromatic disulfide bonds in the system. These reversible bonds are the key to confer the material the target 3R functionality.

The liquid mixture was then poured between two glass plates separated by a 2.5- or 1.5-mm rubber (diameter of the rubber depending on the characterization that will be made on the sheet) in order to manually prepare flat sheets for further characterization. Curing cycle was determined for each formulation by preliminary DSC studies (Supplementary Figure S3) and taking into consideration that, for a complete curing, the system should be cured above its T_g_. These conditions are also given in Table 1. The reaction was followed by FTIR and the disappearance of bands corresponding to the epoxy group at 915 cm⁻^1^ (C–O stretching of oxirane ring) and 3056 cm⁻^1^ (C–H stretching of oxirane ring) confirmed its completion (Supplementary Figure S4).

## 3. Results and Discussion

### 3.1. Thermal and Mechanical Characterization of the Pristine Material

The biobased epoxy vitrimers were prepared by the reaction of the biobased epoxy monomers with the aromatic disulfide based dynamic hardener 4-AFD, as reported in the experimental section. Their thermal and mechanical properties were analyzed, and the results are shown in Table 2.

#### 3.1.1. Thermal Properties

The T_g_ of the prepared vitrimers was determined by DSC (half-height of the heat flow step) and DMA (as the peak of the tan δ curve) analyses (Supplementary Figures S5 and S6). An increasing content of PHTE (trifunctional epoxy resin) in the resin formulation led to an increase in the value of the T_g_ of the prepared material (Table 2). ECO-1 formulation, which is 100% DGEVA (bifunctional epoxy resin), gives a material with a similar T_g_ to the one shown by the reference formulation ECO-REF (105 °C and 107 °C respectively), showing that the developed biobased epoxy resins are comparable to those that are commercially available. In addition, depending on the requirements of the target application, the resin can be formulated with a determined DGEVA:PHTE ratio for reaching an intended T_g_ between 100 and 200 °C.

Degradation temperatures (given as the temperature at which the material has lost 5% of its weight, T_d_5%) are on the same range for all the prepared formulations, either with the reference resin or with the synthesized resins (Supplementary Figure S7).

#### 3.1.2. Mechanical Properties

Mechanical properties of the formulations prepared using the biobased DGEVA and PHTE resins in different ratios were shown to be comparable to, or even improved upon, those of the materials prepared with the reference biobased resin (Supersap CLR) (Table 2—data available in Supplementary Figure S8). These values are also comparable to the mechanical properties of the commonly used DGEBA based thermoset materials [19,20]. As it can be observed, formulations with mixed monomers outperform those with single monomers. The addition of PHTE to the formulation in the lowest ratio (ECO-2), improves the mechanical properties of the obtained material. However, further addition of PHTE does not seem to result in additional increase in stress and strain at break values of the vitrimer. Thus, other factors, such as the crosslink density of the material, may be affecting the mechanical properties.

#### 3.1.3. Crosslink Density

The crosslink density (ν_XL_)of the cured vitrimers were calculated by DMA, according to the theory of rubber elasticity [21] as shown in Equation (1). Results given in Table 2 show that the crosslink density increases with an increasing content in PHTE of the formulation, similarly to the observation made on T_g_ values. An increasing crosslink density may lead to a more brittle material. However, the mechanical properties show that the developed materials improve the properties of the reference material in all cases (with stress values that go from 86 to 105 MPa).

E’ (at Tg + 30 °C) = 3ν_XL_RT (1)

#### 3.1.4. Dynamic Properties

As it has been previously shown, these formulations have similar or even enhanced thermal and mechanical properties compared with the reference formulation (ECO-REF). Additionally, a distinguishing property of the formulations prepared with 4-AFD is that the resulting materials show a dynamic behavior. This is attributed to the aforementioned reversible disulfide bridges that, under determined operational conditions, can rearrange giving the materials the ability of being reprocessable, recyclable and repairable (3R).

Relaxation time is defined as the time required for relaxing 63% of the applied stress. Time and temperature dependent relaxation modulus was measured by DMA (data available in Supplementary Figure S9), to characterize the heat induced malleability and confirm the dynamic behavior of our material prior the reprocessability, recyclability and repairability tests. The stress-relaxation study shows that at temperatures above the T_g_, the developed biobased epoxy networks are able to completely relax stress and flow. Table 3 displays the relaxation times of the prepared materials at different temperatures.

**Figure 2:**
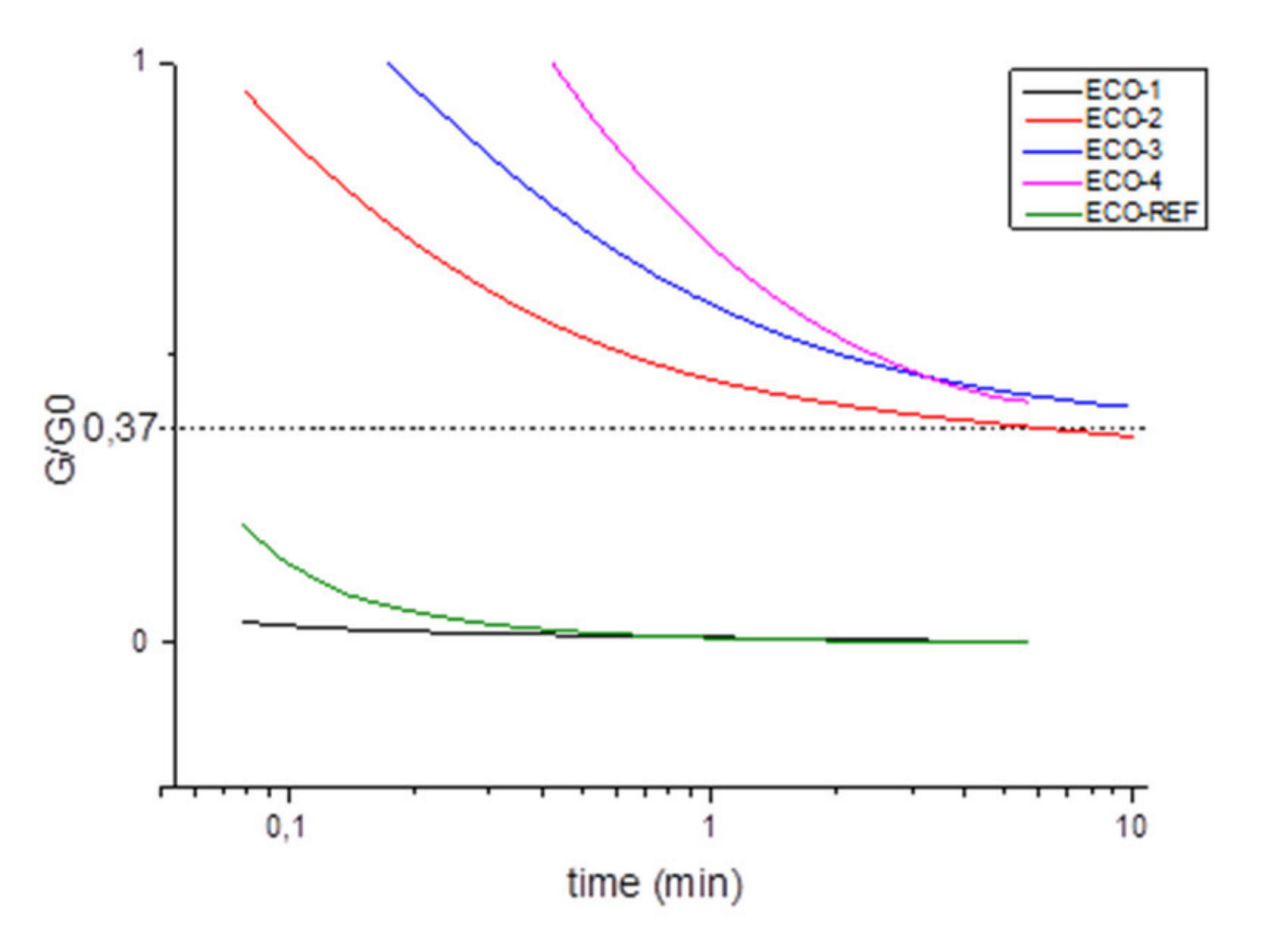
Dynamic behavior of each formulation at 200 °C.

Figure 2 shows the relaxation behavior of all the prepared materials at 200 °C. The results show that the relaxation time clearly depends on the T_g_ of the material and its crosslink density. Samples with the lowest T_g_ values require lower temperatures to relax or have shorter relaxation times at a given temperature (ECO-1 and ECO-REF are completely relaxed at 200 °C).

**Figure 3:**
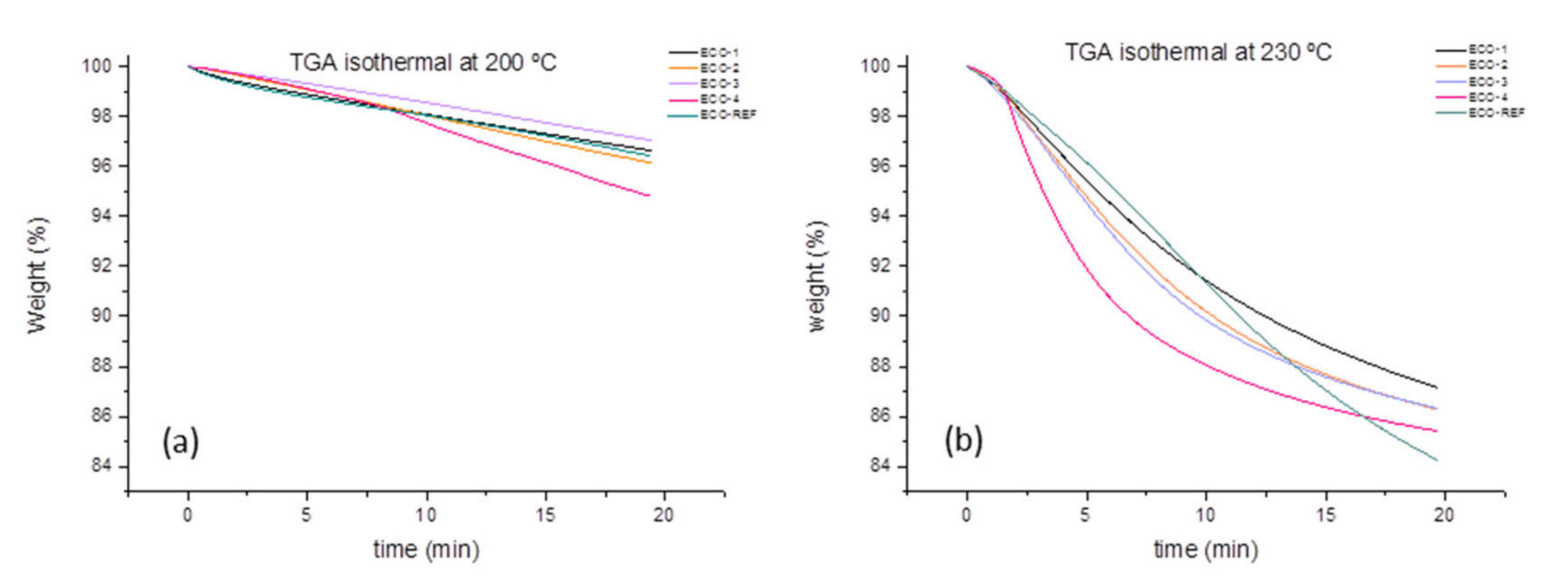
Weight-loss of the prepared samples during the TGA isothermal at: (**a**) 200 °C; and (**b**) 230 °C.

Considering the dependence of the relaxation time and temperature on the T_g_ of the material, TGA isothermal studies were done at 200 and 230 °C to analyze the thermal stability of networks as a function of time. Results (given in Figure 3 and Table 4) show that when the time required to relax the material at a given temperature (above 200 °C) is too long, the degradation of the thermoset may occur due to the thermal degradation of 4-AFD above this temperature.

Figure 3 shows that the weight-loss of the materials after 20 min is similar for all the formulations at each temperature (4–5% at 200 °C and 13–16% at 230 °C).

The thermal degradation, monitored as weight-loss, leads to a decrease of the T_g_ values. In Table 4, the results of the DSC studies performed on the residues of the TGA are provided (data available in Supplementary Figure S10).

Based on the results shown in Table 4, 200 °C was set as the maximum temperature for the recycling/repairing/reprocessing processes, in order to avoid product degradation. Temperature was set at 40–50 °C above the T_g_ of each formulation, at which the relaxation time was around 1–2 min (a little bit higher for the ECO-REF, Table 3). In the case of ECO-4, due to its high T_g_, this material would require working at too high temperatures for at least 30 min to be repaired, recycled, or reprocessed. At these conditions, the material would degrade and, therefore, the study of the 3R properties of this formulation was not possible.

Thus, once the relaxation temperatures were set at 40–50 °C above the T_g_ of the materials, the next step was to test their 3R properties:

- Reprocessability

The developed materials should be easily reprocessable or reshapable above their T_g_, based on their dynamic behavior conferred by the aromatic disulfide bonds in their structure. A hot press and a zig-zag shaped mold were used to change the shape of the materials and demonstrate this fact. After 10 min at the temperature specified for each formulation (40–50 °C above their T_g_) a self-standing zig-zag film was obtained.

**Figure 4:**
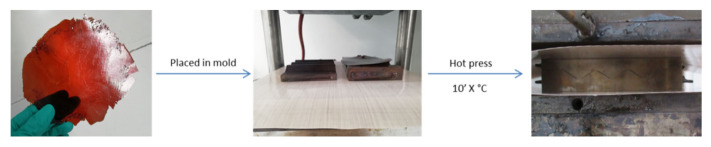
Procedure for reprocessing the prepared materials.

The same procedure (shown in Figure 4) was followed for all the samples: a zig-zag shaped mold and the hot-press were pre-heated to the required temperature (150 °C for ECO-1 and ECO-REF; 180 °C for ECO-2 and 200 °C for ECO-3). Once the entire system was tempered, a cured film of the corresponding formulation was placed in the mold in the hot press. Heat and pressure (100 bar) were applied for 10 min so the material has enough time to adapt to the new shape. The mold was let to cool down to room temperature, closed but unloaded.

**Figure 5:**
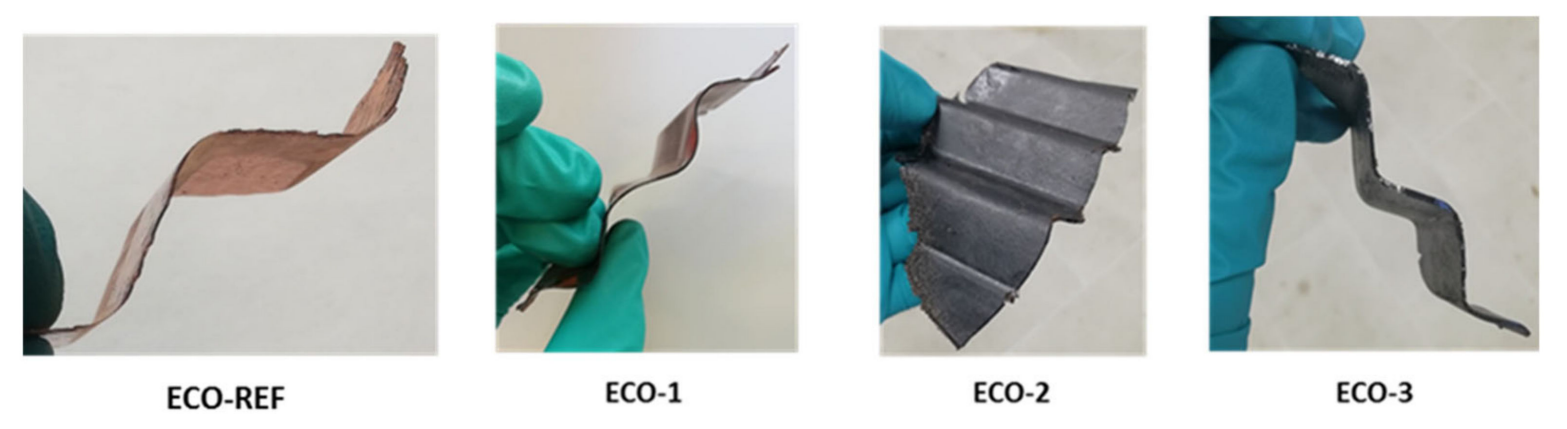
Reprocessed materials after 10 min at: 150 °C (ECO-1 and ECO-REF); 180 °C (ECO-2), and 200 °C (ECO-3).

This process led in all cases to new 3D parts of the previously prepared and cured biobased epoxy thermosets, as shown in Figure 5.

- Repairability

**Figure 6:**
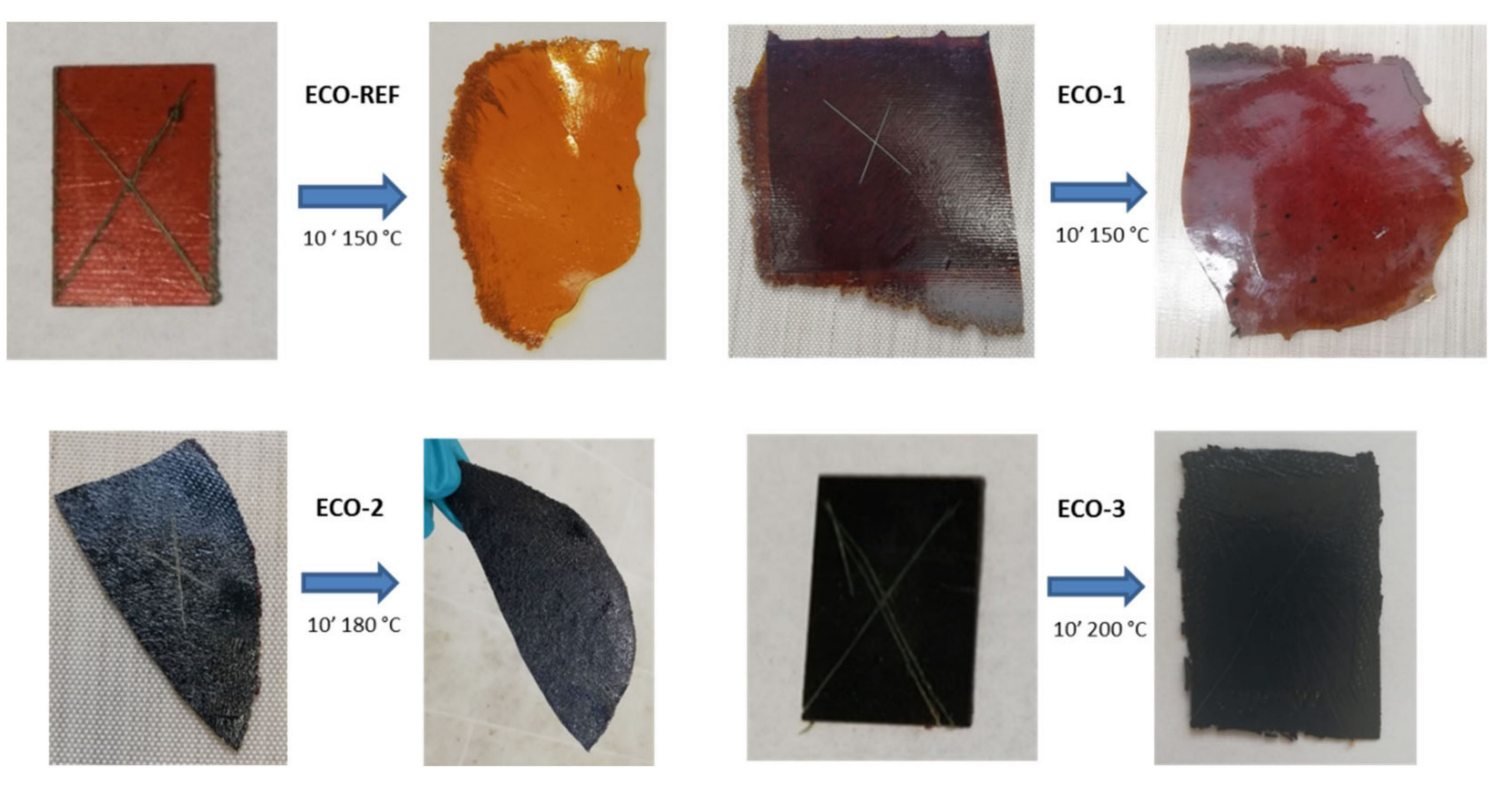
Repairing of the prepared materials by applying heat and pressure for 10′ at: 150 °C (ECO-1); 180 °C (ECO-2); and 200 °C (ECO-3).

During service, thermosets may suffer different kinds of damage. Current repair approaches require special techniques and skilled workers, making this possibility slow and expensive. The developed dynamic biobased thermosets can be repaired just applying heat and pressure for a short time (Figure 6). Here again, the temperature will depend on the relaxation times given by each formulation (set at a temperature 40–50 °C over their corresponding T_g_; 150 °C for ECO-REF and ECO-1, 180 °C for ECO-2, and 200 °C for ECO-3).

To show that these materials are repairable, damage was caused on purpose on the surface of a cured film (a cross was made on them with a scalpel). This film was placed then inside the hot-press (in between two Teflon films), which had been pre-heated to the corresponding temperature. The films were left inside the closed hot-press (with no external pressure) for 10 min and the disappearance of the cross could be observed when taking them out of the press, as shown in Figure 6. The films are flattened due to the pressure put on them by the hot-press.

- Reciclability

**Figure 7:**
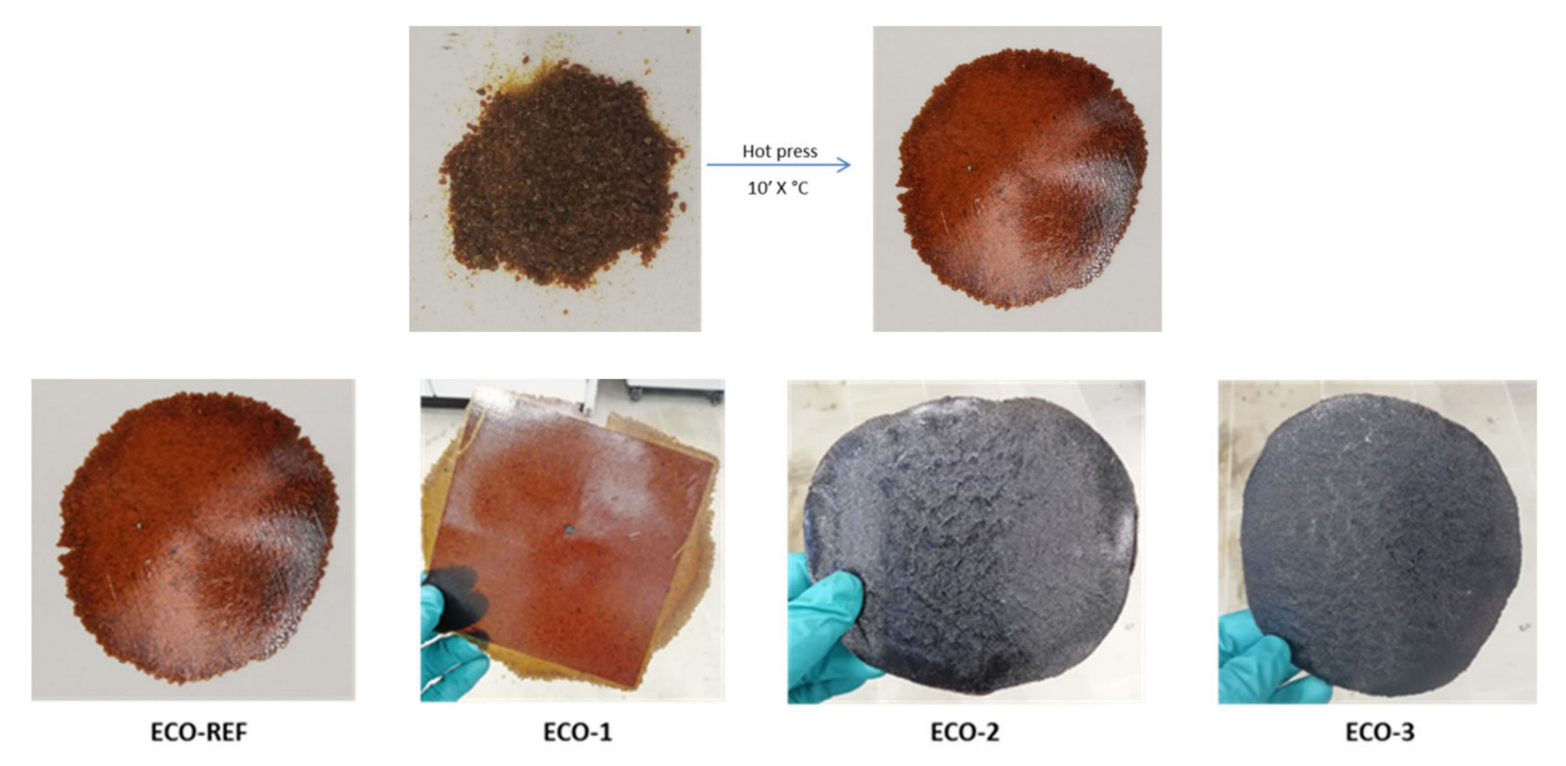
Recycled materials after 10′ at: 150 °C (ECO-1); 180 °C (ECO-2), and 200 °C (ECO-3).

Thermoset materials are not easily recyclable. In contrast to this, the biobased vitrimers developed in this work can be recycled by a simple process of mechanical recycling and subsequent thermoforming. To show this, the cured sheets were grinded to powder and this powder was then hot pressed for 10 min at a temperature 40–50 °C over its T_g_ in order to obtain a new sheet (Figure 7).

The thermomechanical properties of these sheets were characterized in order to check that they did not change compared to the pristine sheets. Results are described below.

### 3.2. Thermal and Mechanical Characterization of the Recycled Material

#### Thermomechanical Properties Recovery

The thermal properties of the recycled materials were tested by DSC (T_g_), DMA (T_g_) and TGA (T_d_5%) and results are shown in Table 5. Recycled ECO-1 and ECO-REF showed similar T_g_ values to those of the pristine materials. However, in ECO-2 and ECO-3 an increase of the T_g_ value is observed, probably promoted by a “post-curing” step induced by the recycling process.

Storage modulus (E’) of the recycled materials was measured by DMA as a rough estimate of their mechanical properties, due to the fact that the recycled samples were not big enough for obtaining the specimens required for tensile tests in the Instron (as it was done for the pristine materials). Results show (Table 6) that there is a slight variation on the properties of the recycled materials when compared to their pristine counterparts. Nevertheless, they are still comparable to those of the commonly used DGEBA based thermosets [19,20].

## 4. Conclusions

In the present paper, we highlight the synthesis of reprocessable, repairable and recyclable biobased epoxy vitrimers from DGEVA and PHTE biobased epoxy resins and 4-AFD as dynamic hardener. This combination of resin/hardener couple lead to the formation of epoxy vitrimers with fast relaxation times. It is demonstrated here that the thermo-mechanical properties of the thermoset materials can be adjusted to the requirement of a given application by mastering the ratio of DGEVA/PHTE epoxy resins. Indeed, T_g_ values in between 100 and 200 °C could be obtained. Furthermore, the thermal and mechanical properties of developed materials are similar to those of commonly used DGEBA based thermosets [19,20] and those of the reference epoxy formulations selected in this study (Supersap CLR epoxy resin). Finally, the approach proposed in this paper appears as a relevant technical solution to solve the main drawbacks linked to epoxy thermoset materials: (i) the use of non-renewable fossil-derived precursors is avoided; (ii) toxic BPA is no longer used; and (iii) developed materials can be efficiently repaired, reprocessed, and recycled.

## Figures and Tables

**Table 1:** Formulations and curing cycle of the prepared epoxy vitrimers.

Sample Name	DGEVA/PHTE Ratio (wt)	DGEVA	PHTE	SUPERSAP CLR	4-AFD	Curing Cycle
ECO-1	100/0	30 g, 0.22 epoxy eq.	-	-	16.14 g, 0.26 amino eq.	1 h 150 °C
ECO-2	60/40	18 g, 0.13 epoxy eq.	12 g, 0.08 epoxy eq.	-	15.52 g, 0.25 amino eq.	1 h 150 °C
ECO-3	40/60	12 g, 0.09 epoxy eq.	18 g, 0.12 epoxy eq.	-	15.52 g, 0.25 amino eq.	1 h 160 °C
ECO-4	0/100	-	30 g, 0.20 epoxy eq.	-	14.90 g, 0.24 amino eq.	1 h 150 °C + 30′ 180 °C
ECO-REF	-	-	-	30 g, 0.16 epoxy eq.	11.80 g, 0.19 amino eq	1 h 150 °C

**Table 2:** Thermomechanical properties of the prepared formulations.

Ref.	T_g_^DSC^ (°C)	T_g_^DMA^ (°C)	T_d_5% (°C)	Stress ^a^ (MPa)	Strain ^a^ (%)	E’ (30 °C) (GPa)	E’ (at T_g_ + 30 °C) (MPa)	ν_XL_^b^·10^3^ (mol·cm^−3^)
ECO-1	105	103	259	86 ± 1	7 ± 1	6	17	1.7
ECO-2	135	137	251	105 ± 2	7 ± 0.6	9	28	2.6
ECO-3	157	160	257	94 ± 4	5 ± 0.4	7	41	3.6
ECO-4	194	197	239	92 ± 6	5 ± 0.3	11	83	6.7
ECO-REF	107	108	252	66 ± 10	4 ± 1	7	16	1.6

^a^ Values at break. ^b^ ν_XL_: crosslink density calculated by DMA according to the theory of rubber elasticity [21].

**Table 3:** Relaxation times at different temperatures ((T_g_ + 20 °C) and (T_g_ + 50 °C)) for the prepared materials.

Ref.	Relaxation Time (s)	
	T_g_ + 20 °C	T_g_ + 50 °C
ECO-1	It does not relax (at 120 °C)	100 s (at 150 °C)
ECO-2	It does not relax (at 150 °C)	163 s (at 180 °C)
ECO-3	215 s (at 170 °C)	35 s (at 200 °C)
ECO-4	359 s (at 210 °C–too high T)	Too high T (240 °C)
ECO-REF	It does not relax (at 120 °C)	212 s (at 150 °C)

**Table 4:** DSC analysis of the prepared materials before and after the TGA isothermal for 20 min at 200 °C and 230 °C.

Ref.	Initial T_g_^DSC^ (°C)	T_g_^DSC^ after 20 min at 200 °C (°C)	T_g_^DSC^ after 20 min at 230 °C (°C)
ECO-1	105	97	95
ECO-2	134	131	129
ECO-3	156	152	137
ECO-4	194	177	162
ECO-REF	107	102	92

**Table 5:** Thermal properties of the prepared vitrimers (pristine and recycled).

Ref.	Initial T_g_^DSC^ (°C)	Recycled T_g_^DSC^ (°C)	Initial T_g_^DMA^ (°C)	Recycled T_g_^DMA^ (°C)	Initial T_d_5^%^ (°C)	Recycled T_d_5^%^ (°C)
ECO-1	105	106	103	103	259	255
ECO-2	135	149	137	146	251	255
ECO-3	157	165	160	169	257	255
ECO-REF	107	104	108	107	252	249

**Table 6:** Mechanical properties of the prepared vitrimers (pristine and recycled).

Ref.	Initial E’ (30 °C) (GPa)	Recycled E’ (30 °C) (GPa)	Initial E’ (T_g_ + 30 °C) (MPa)	Recycled E’ (T_g_ + 30 °C) (MPa)	Initial ν_XL_^a^·10^3^ (mol·cm^−3^)	Recycled ν_XL_^a^·10^3^ (mol·cm^−3^)
ECO-1	6	4	17	20	1.7	2.0
ECO-2	9	10	28	26	2.6	2.3
ECO-3	7	4	41	54	3.6	4.6
ECO-REF	7	3	16	16	1.6	1.6

a ν_XL_: crosslink density calculated by DMA according to the theory of rubber elasticity [21].

## Supplementary Materials

The following are available online at https://www.mdpi.com/2073-4360/12/11/2645/s1. Figure S1: ^1^H NMR spectra of DGEVA—NMR 300MHz—CDCl3, Figure S2: ^1^H NMR spectra of PHTE—NMR 300MHz—Acetone-d_6_, Figure S3: DSC thermograms for all the prepared formulations from which curing conditions were determined, Figure S4: FTIR spectra of uncured (black trace) and cured (red trace) epoxy resin systems. The disappearance of bands corresponding to the epoxy group at 915 cm⁻^1^ (C–O stretching of oxirane ring) and 3056 cm⁻^1^ (C–H stretching of oxirane ring) was used as criteria to establish that the curing was complete, Figure S5: DSC thermograms for all the prepared epoxy networks from which the T_g_ values were determined for each formulation: 105 °C for ECO-1; 135 °C for ECO-2; 157 °C for ECO-3; 194 °C for ECO-4; and 107 °C for ECO-REF. It can be observed the absence of other exothermic peaks associated to residual curing, Figure S6: DMA curves obtained for the prepared vitrimers, representing storage modulus and tan delta versus temperature. T_g_ values were determined for each formulation from the maximum of tan delta: 103 °C for ECO-1; 137 °C for ECO-2; 160 °C for ECO-3; 197 °C for ECO-4; and 108 °C for ECO-REF. E’ (30 °C) and E’ (T_g_ + 30 °C) values were also determined from these curves: 6.10^3^ MPa and 17 MPa for ECO-1; 9.10^3^ MPa and 28 MPa for ECO-2; 7.10^3^ MPa and 41 MPa for ECO-3; 11.10^3^ MPa and 83 MPa for ECO-4; and 7.10^3^ MPa and 16 MPa for ECO-REF (for E’ (30 °C) and E’ (T_g_ + 30 °C) respectively), Figure S7: TGA thermogram of the prepared vitrimers representing the weight loss versus temperature. T_d_(5%) was determined for each formulation as the temperature at which the material has lost 5% of its weight: 259 °C for ECO-1; 251 °C for ECO-2; 257 °C for ECO-3; 239 °C for ECO-4; and 252 °C for ECO-REF, Figure S8: Stress-strain curves for the prepared epoxy networks, Figure S9: Normalized stress relaxation curves for all the prepared vitrimers at different temperatures from which their relaxation times were determined at temperatures 20 °C and 50 °C above their T_g_, Figure S10: DSC thermograms for the residues obtained after the TGA isothermals (20 min) at 200 °C and 230 °C for all the prepared vitrimers. T_g_ values after these isothermals were determined and compared to the T_g_ of the pristine epoxy for each formulation: 105 °C, 97 °C and 95 °C for ECO-1 (untreated, after 20 min at 200 °C and after 20 min at 230 °C respectively); 135 °C, 131 °C and 129 °C for ECO-2 (untreated, after 20 min at 200 °C and after 20 min at 230 °C respectively); 157 °C, 152 °C and 137 °C for ECO-3 (untreated, after 20 min at 200 °C and after 20 min at 230 °C respectively); 194 °C, 177 °C and 162 °C for ECO-4 (untreated, after 20 min at 200 °C and after 20 min at 230 °C respectively); and 107 °C, 102 °C and 92 °C for ECO-REF (untreated, after 20 min at 200 °C and after 20 min at 230 °C respectively).
