# Supplementary material for: Build-To-Specification Vanillin and Phloroglucinol Derived Biobased Epoxy-Amine Vitrimers
Source: Polymers (Basel). 2020 Nov 10;12(11):2645. doi: 10.3390/polym12112645 (PMC7697130; doi:10.3390/polym12112645)
Supplement: Supplementary file 1 [file polymers-12-02645-s001.pdf]

Electronic Supplementrary Information

Build-to-specification vanillin and phloroglucinol derived biobased epoxy-amine vitrimers

Aratz Genua,[*,1], Sarah Montes,[1] Itxaso Azcune,[1] Samuel Malburet,[2] Bénédicte Daydé-Cazals,[2] Alain Graillot[2]

[1] CIDETEC, Basque Research and Technology Alliance (BRTA), Paseo Miramón, 196, 20014 Donostia-San Sebastián, Spain; smontes@cidetec.es; iazcune@cidetec.es.

[2] SPECIFIC POLYMERS, Zac Via Domita, 150 Avenue des Cocardières, 34160 Castries, France; samuel.malburet@specificpolymers.fr; benedicte.dayde-cazals@specificpolymers.fr; alain.graillot@specificpolymers.fr.

* Correspondence: agenua@cidetec.es; Tel.: +34-943318212

[1]H Nuclear magnetic resonance (NMR)

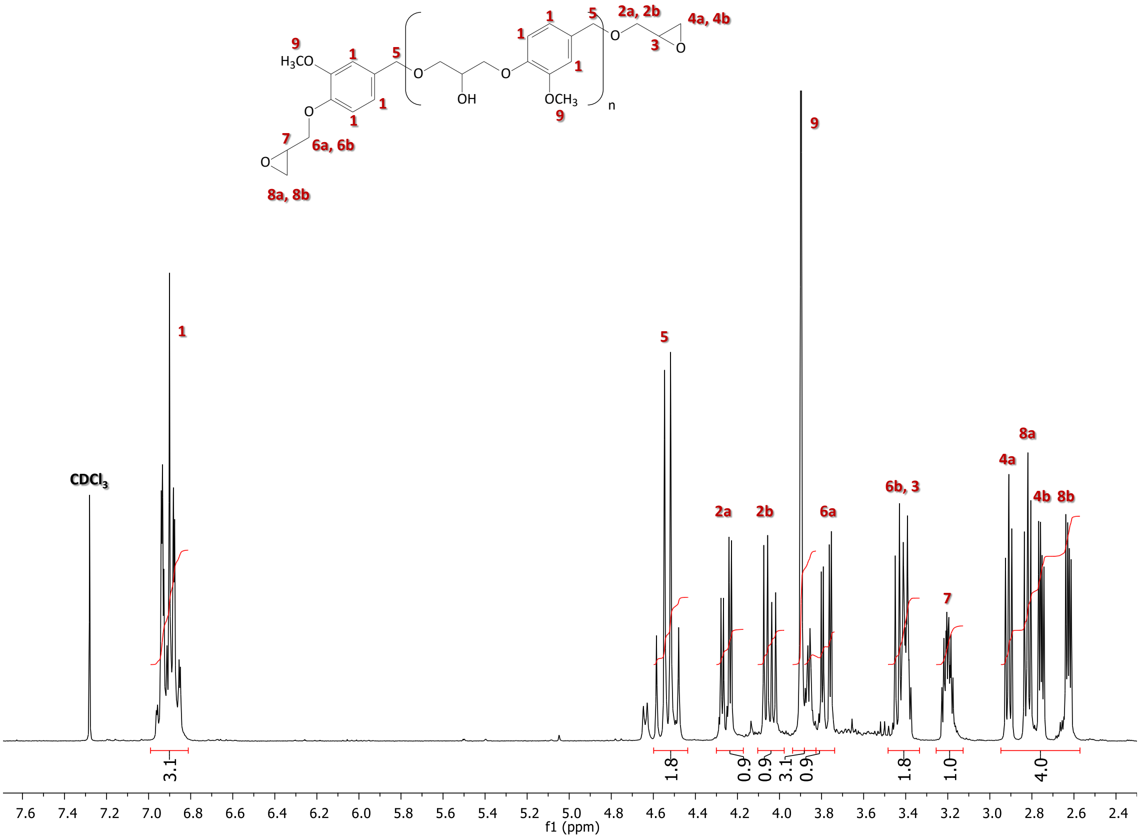

Figure S1: ^{1}H NMR spectra of DGEVA – NMR 300MHz – $CDCl_3$

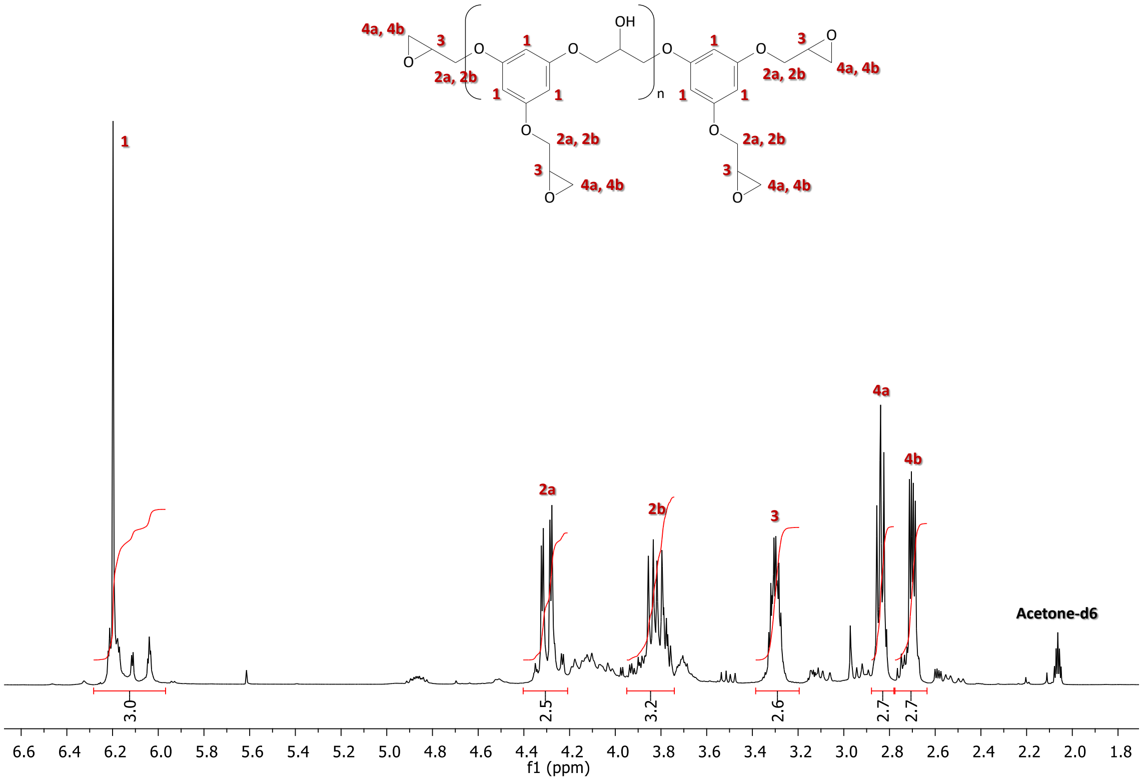

Figure S2: ^{1}H NMR spectra of PHTE – NMR 300MHz – Acetone-d_6

Differential Scanning Calorimetry (DSC) for setting the curing conditions

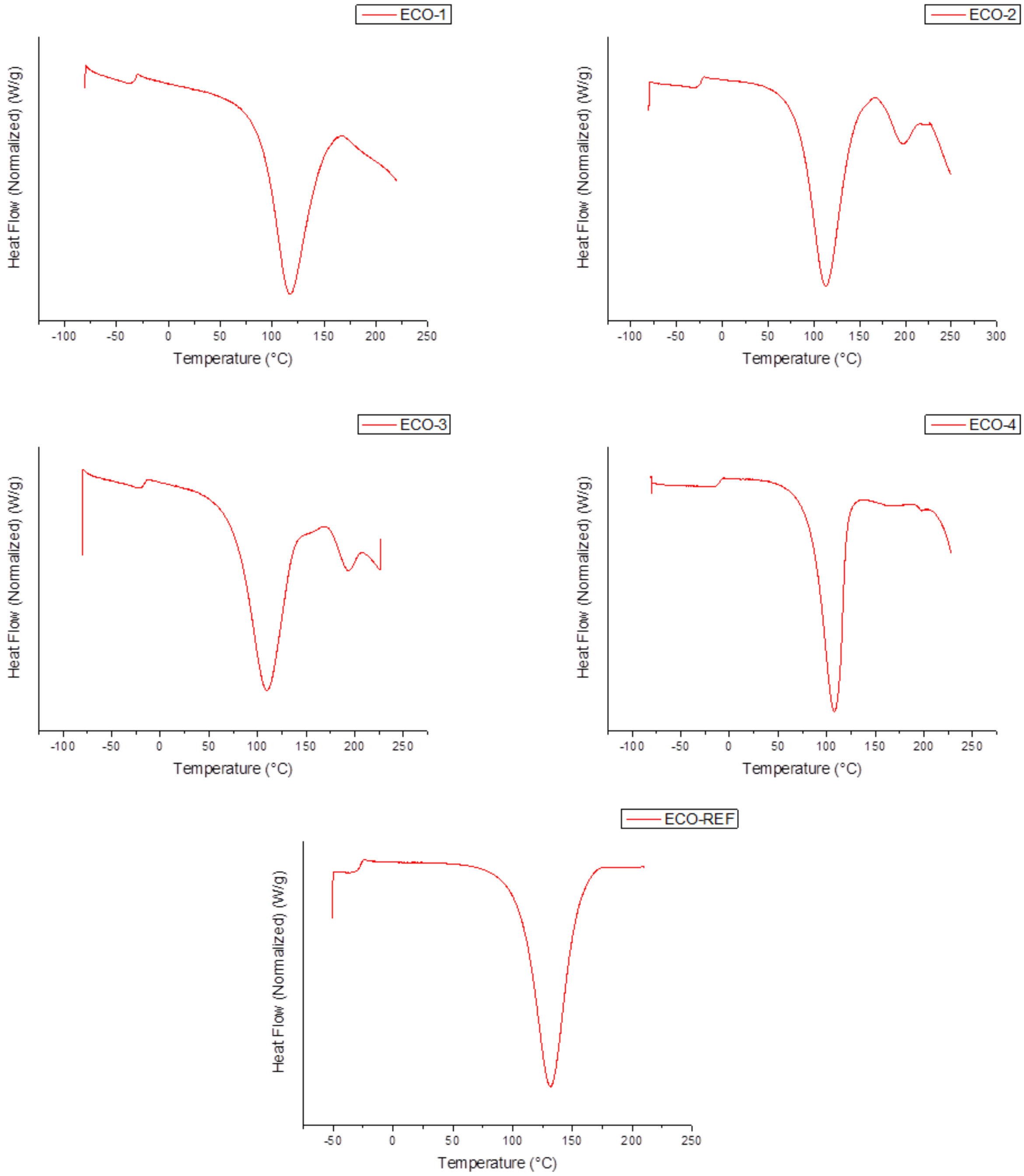

Figure S3: DSC thermograms for all the prepared formulations from which curing conditions were determined.

Fourier-Transform Infrared (FTIR)

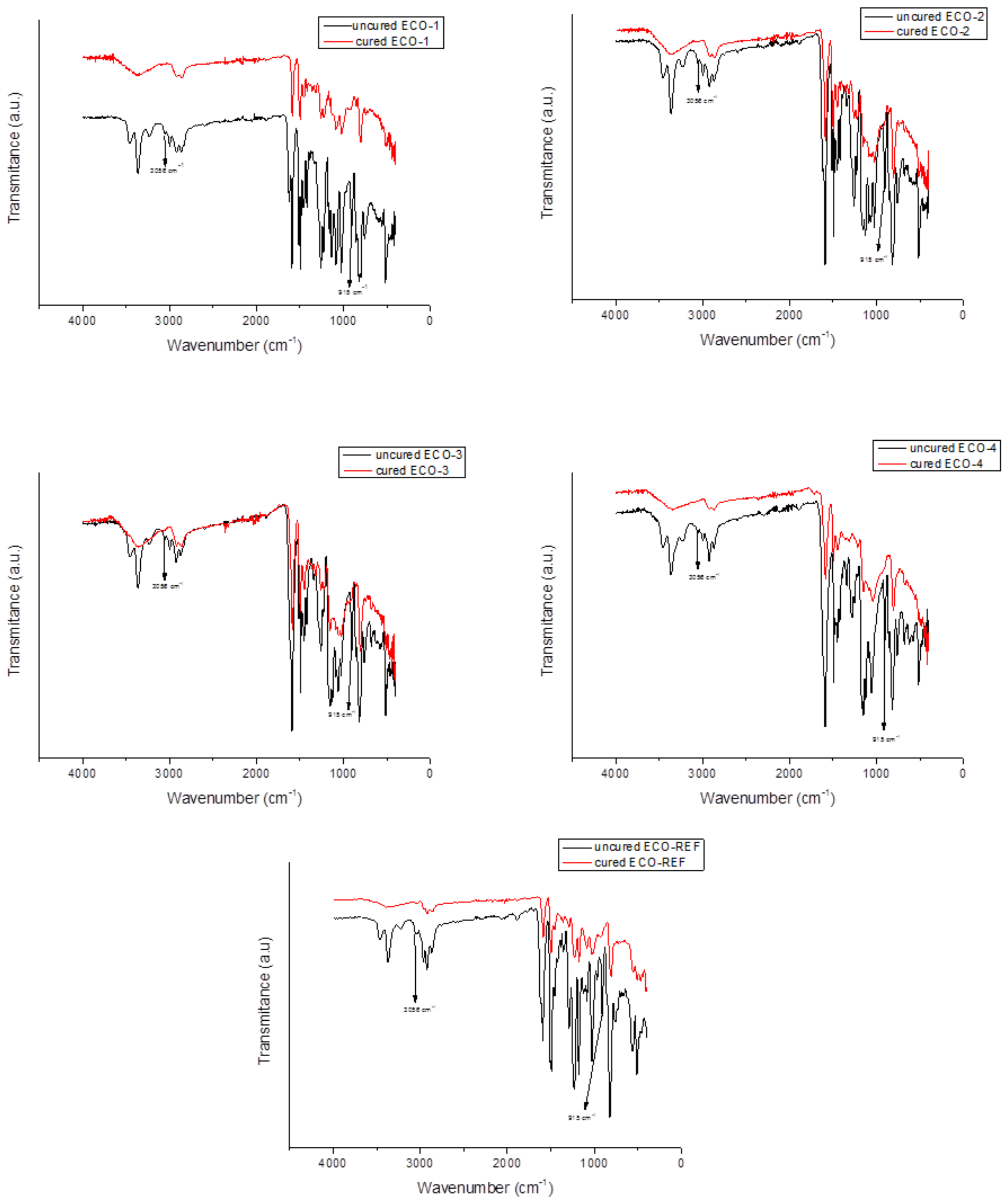

Figure S4: FTIR spectra of uncured (black trace) and cured (red trace) epoxy resin systems. The disappearance of bands corresponding to the epoxy group at 915 cm^{-1} (C-O stretching of oxirane ring) and 3056 cm^{-1} (C-H stretching of oxirane ring) was used as criteria to establish that the curing was complete.

DSC for determining the Tg

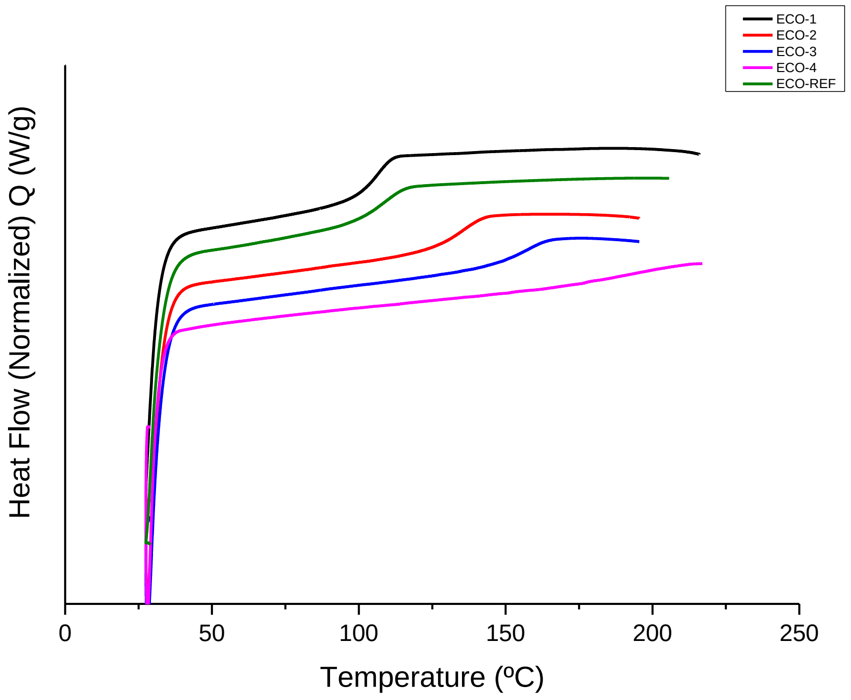

Figure S5: DSC thermograms for all the prepared epoxy networks from which the T_g values were determined for each formulation: 105 °C for ECO-1; 135 °C for ECO-2; 157 °C for ECO-3; 194 °C for ECO-4; and 107 °C for ECO-REF. It can be observed the absence of other exothermic peaks associated to residual curing

Dynamic mechanical analysis (DMA)

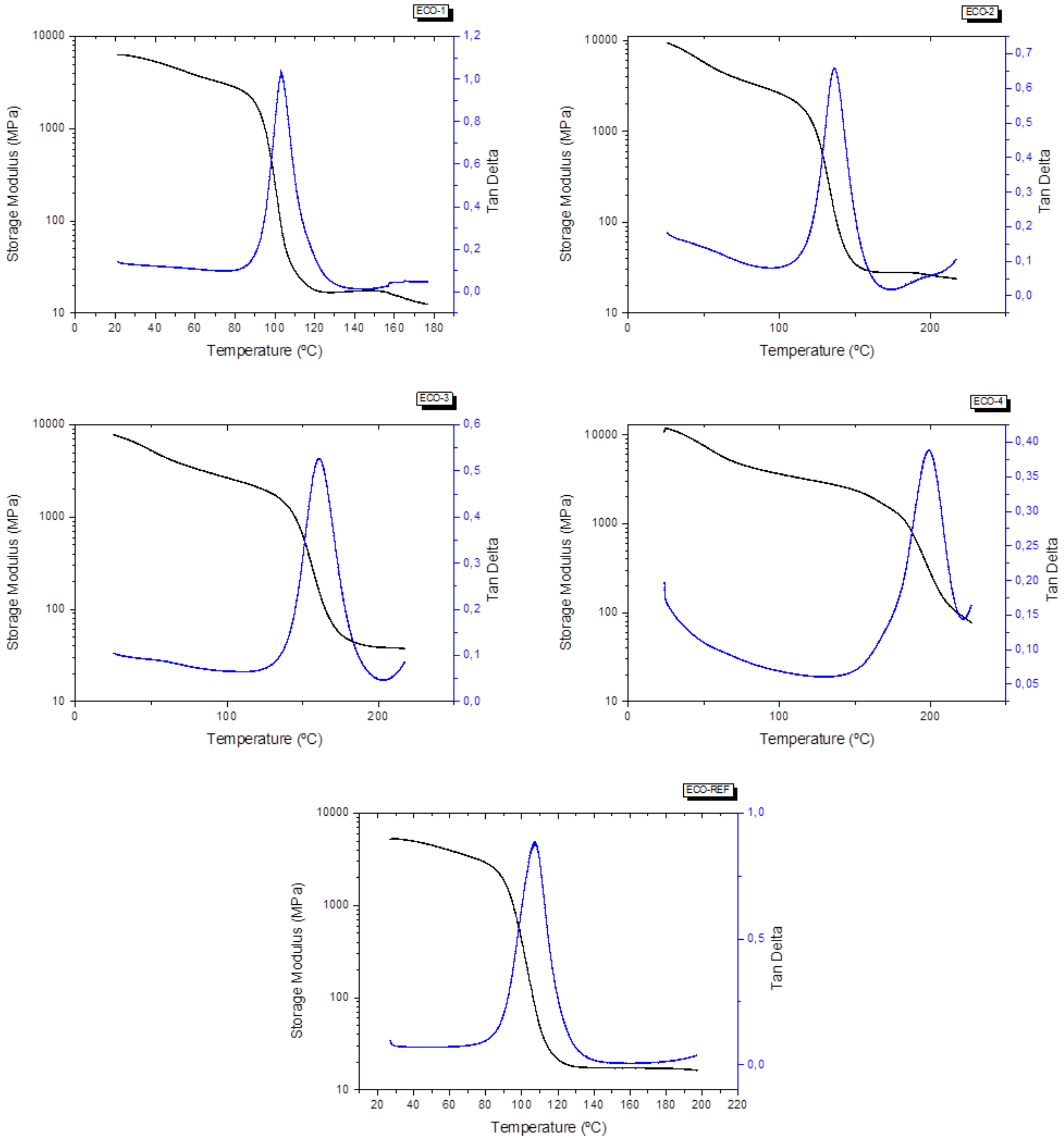

Figure S6: DMA curves obtained for the prepared vitrimers, representing storage modulus and tan delta versus temperature. T_g values were determined for each formulation from the maximum of tan delta: 103 °C for ECO-1; 137 °C for ECO-2; 160 °C for ECO-3; 197 °C for ECO-4; and 108 °C for ECO-REF. E’ (30 °C) and E’ (T_g + 30 °C) values were also determined from these curves: 6.10^3 MPa and 17 MPa for ECO-1; 9.10^3 MPa and 28 MPa for ECO-2; 7.10^3 MPa and 41 MPa for ECO-3; 11.10^3 MPa and 83 MPa for ECO-4; and 7.10^3 MPa and 16 MPa for ECO-REF (for E’ (30 °C) and E’ (T_g + 30 °C) respectively).

Thermogravimetric analysis (TGA)

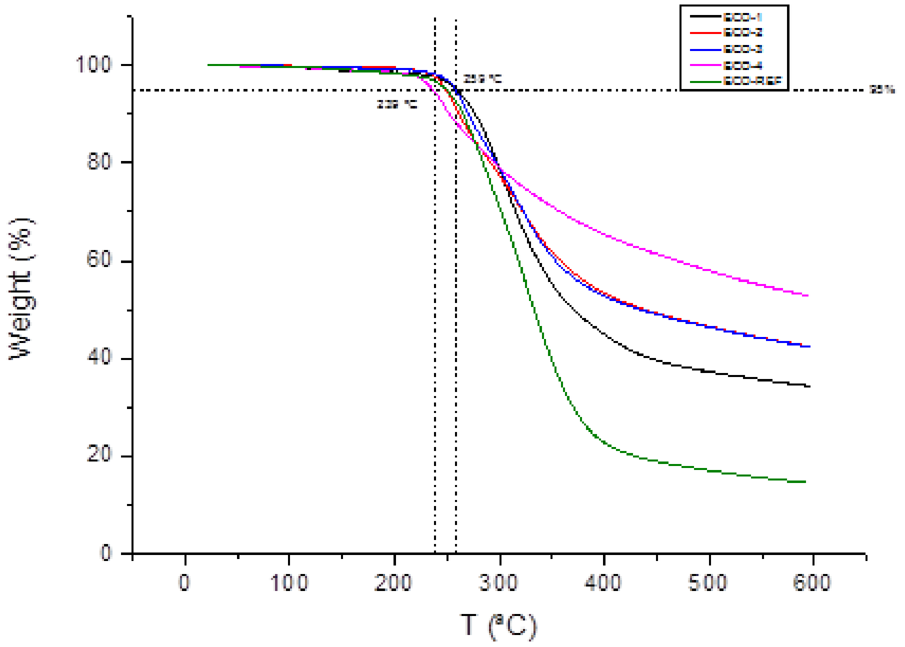

Figure S7: TGA thermogram of the prepared vitrimers representing the weight loss versus temperature. $T_d(5\%)$ was determined for each formulation as the temperature at which the material has lost 5% of its weight: 259 °C for ECO-1; 251 °C for ECO-2; 257 °C for ECO-3; 239 °C for ECO-4; and 252 °C for ECO-REF.

Mechanical tests

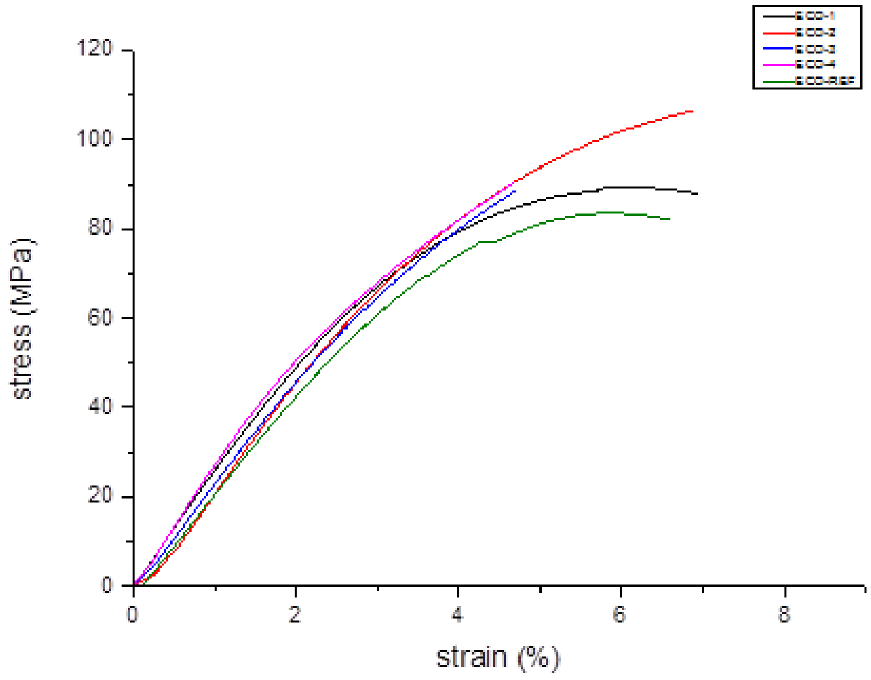

Figure S8: Stress-strain curves for the prepared epoxy networks

Stress relaxation

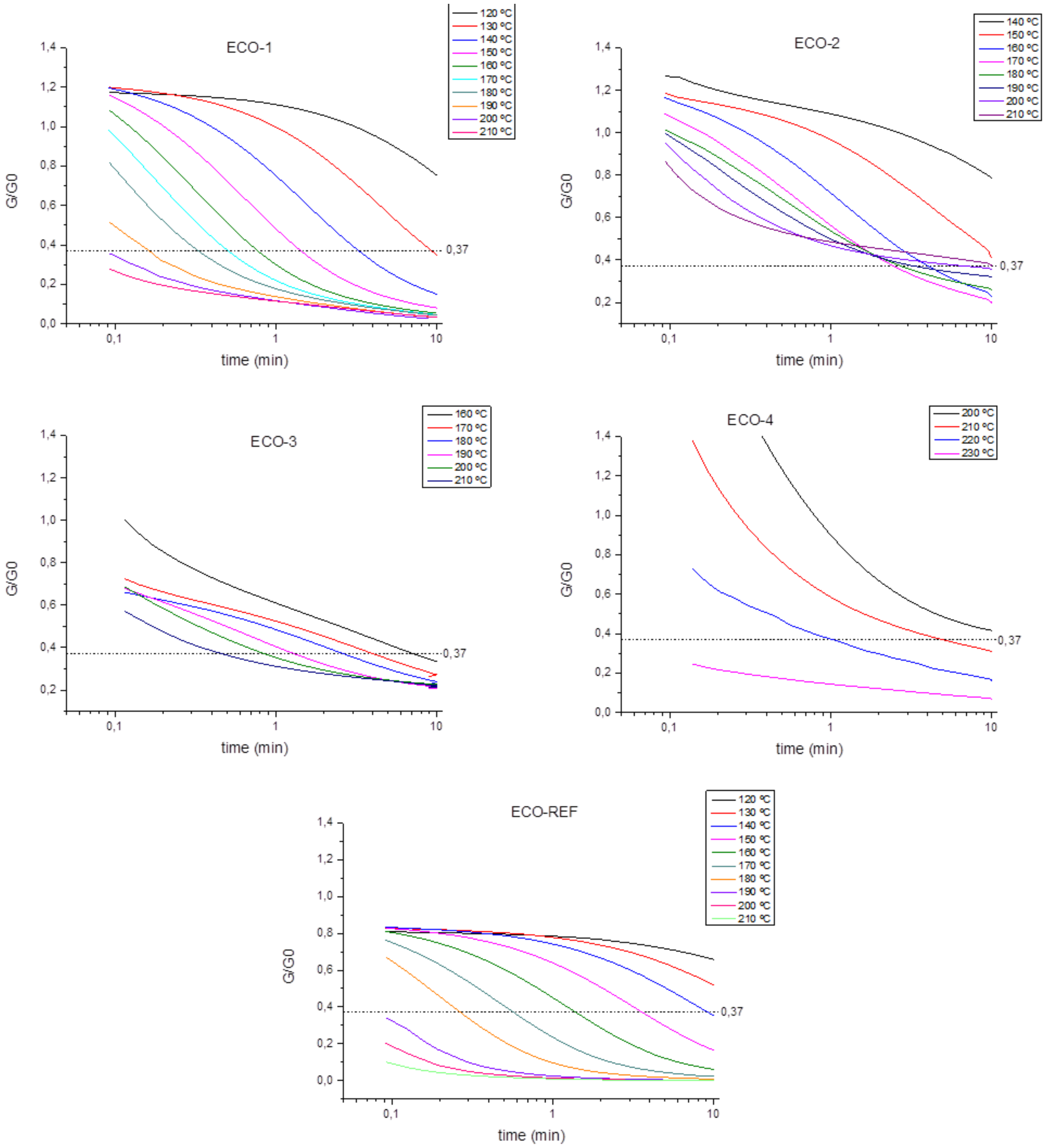

Figure S9: Normalized stress relaxation curves for all the prepared vitrimers at different temperatures from which their relaxation times were determined at temperatures 20 °C and 50 °C above their T_g.

<u>**DSC for determining the Tg of the residues of the TGA isothermals**</u>

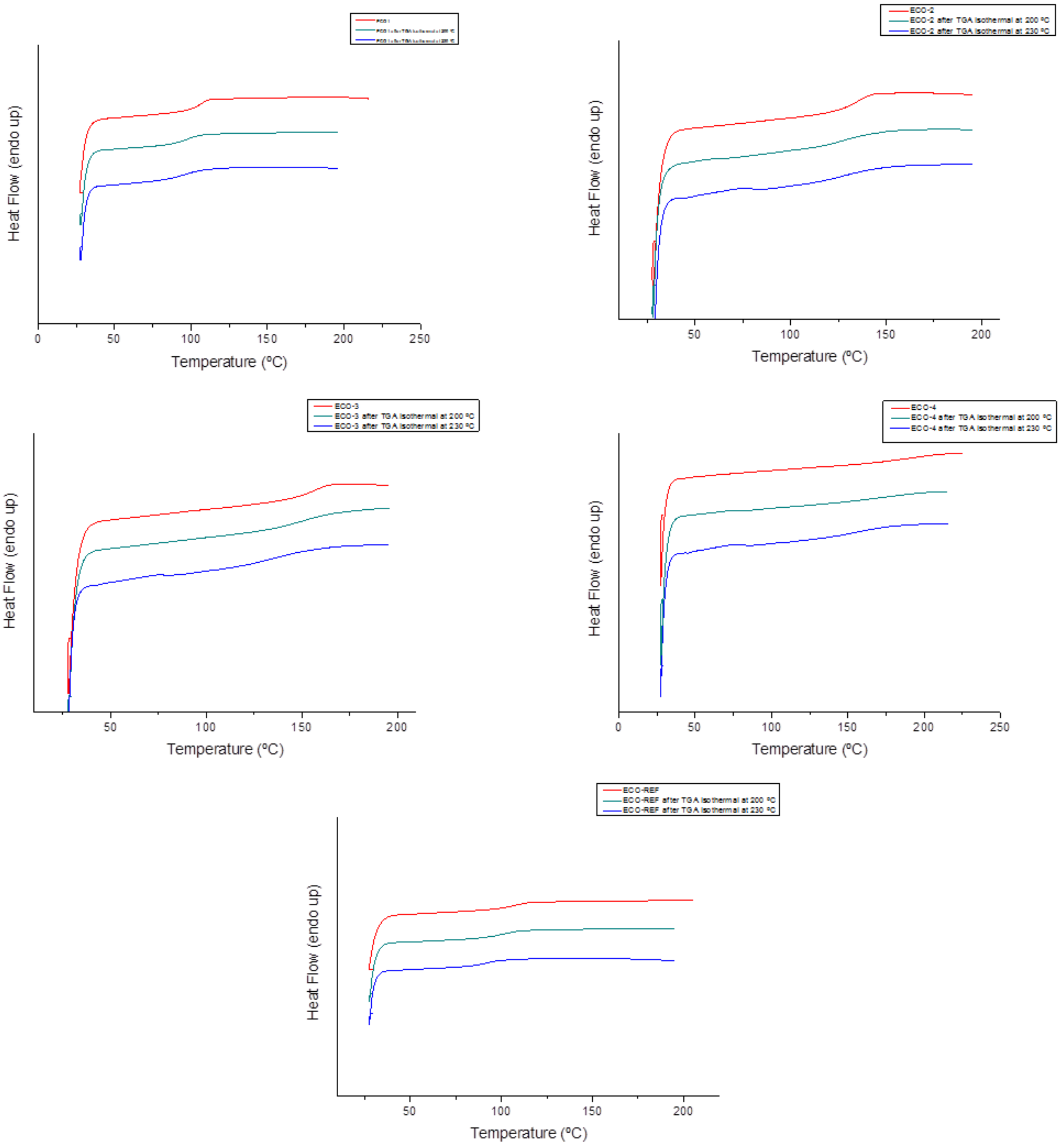

Figure S10: DSC thermograms for the residues obtained after the TGA isothermals (20 min) at 200 °C and 230 °C for all the prepared vitrimers. T_g values after these isothermals were determined and compared to the T_g of the pristine epoxy for each formulation: 105 °C, 97 °C and 95 °C for ECO-1 (untreated, after 20 min at 200 °C and after 20 min at 230 °C respectively); 135 °C, 131 °C and 129 °C for ECO-2 (untreated, after 20 min at 200 °C and after 20 min at 230 °C respectively); 157 °C, 152 °C and 137 °C for ECO-3 (untreated, after 20 min at 200 °C and after 20 min at 230 °C respectively); 194 °C, 177 °C and 162 °C for ECO-4 (untreated, after 20 min at 200 °C and after 20 min at 230 °C respectively); and 107 °C, 102 °C and 92 °C for ECO-REF (untreated, after 20 min at 200 °C and after 20 min at 230 °C respectively).
